# Supplementary material for: X-linked deletion of Crossfirre, Firre, and Dxz4 in vivo uncovers diverse phenotypes and combinatorial effects on autosomes
Source: Nat Commun. 2024 Dec 5;15:10631. doi: 10.1038/s41467-024-54673-5 (PMC11621363; doi:10.1038/s41467-024-54673-5)
Supplement: Supplementary file 1 — Supplementary Information [file 41467_2024_54673_MOESM1_ESM.pdf]

Supplementary Information

X-linked deletion of *Crossfirre*, *Firre*, and *Dxz4 in vivo* uncovers diverse phenotypes and combinatorial effects on autosomes

Tim P. Hasenbein*, Sarah Hoelzl*, Zachary D. Smith, Chiara Gerhardinger, Marion O. C. Gonner, Antonio Aguilar-Pimentel, Oana V. Amarie, Lore Becker, Julia Calzada-Wack, Nathalia R. V. Dragano, Patricia da Silva-Buttkus, Lillian Garrett, Sabine M. Hölter, Markus Kraiger, Manuela A. Östereicher, Birgit Rathkolb, Adrián Sanz-Moreno, Nadine Spielmann, Wolfgang Wurst, Valerie Gailus-Durner, Helmut Fuchs, Martin Hrabe de Angelis, Alexander Meissner, Stefan Engelhardt, John L. Rinn† and Daniel Andergassen†

* Authors contributed equally to this work
† Correspondence

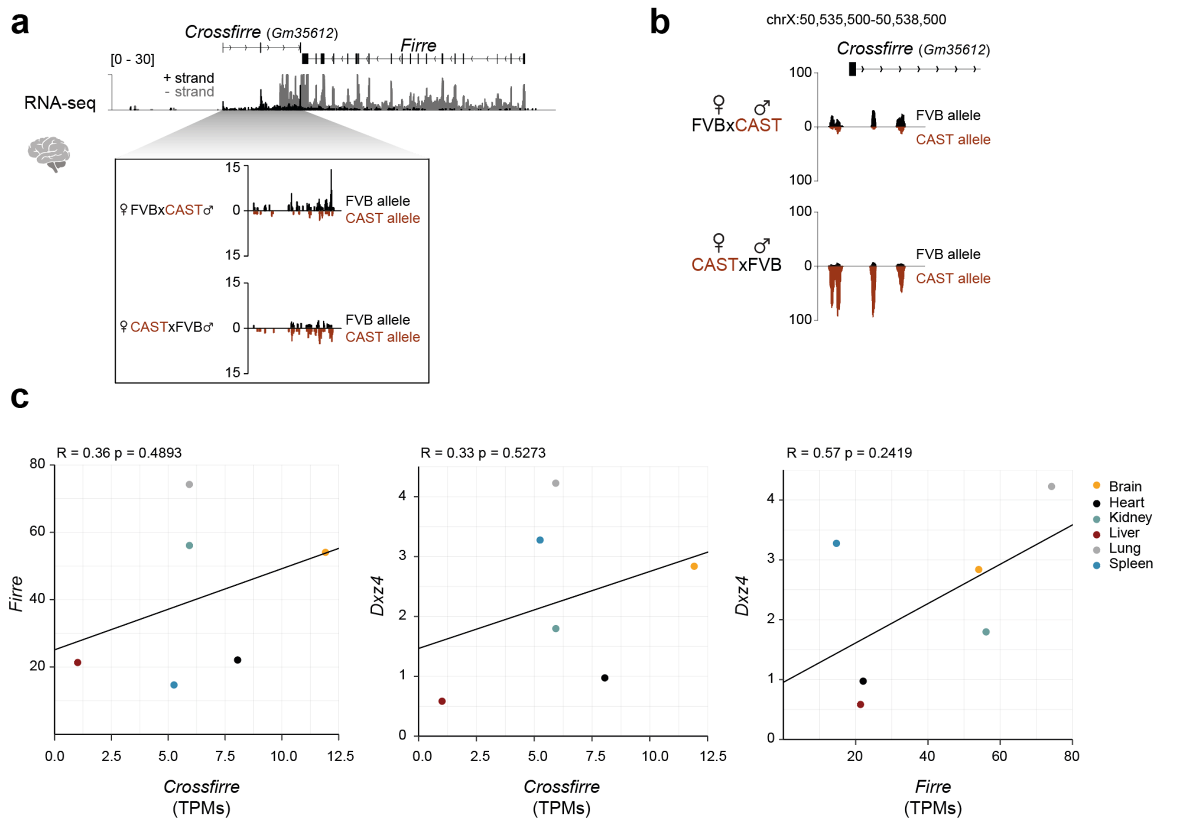

Supplementary Fig. 1: Imprinting of the *Crossfirre* locus and correlation analysis of *Crossfirre*, *Firre* and *Dxz4*.
a, Genome browser track of strand-specific RNA-seq data from female F1 brain covering the *Crossfirre* (*Gm35612*) and *Firre* locus[15]. The zoom out below shows the aligned forward sequencing reads of *Crossfirre* after allele-specific splitting using SNPsplit[53]. Sequencing reads originating from the FVB and CAST allele are indicated in black and brown, respectively. The cross scheme is depicted next to the browser track. **b**, Allele-specific splitting of H3K4me3 sequencing reads covering the *Crossfirre* promoter in female mouse embryonic fibroblasts (genetic background as in a). Sequencing reads originating from the FVB and CAST allele are indicated in black and brown, respectively. Publicly available data was used from [54]. **c,** Pearson's correlation of mean TPM values between *Crossfirre*, *Firre* and *Dxz4* (*n* = 4 per tissue).

a

1	gRNA_Crossfirre_up	GATCTTTACCCCACAGTATAAGG
2	gRNA_Crossfirre_down	GGGATGGCCACACCTCACAATGG
3	gRNA_Crossfirre-Firre_up	AATGGGTCCAGGTATTGGCGGGG
4	gRNA_Crossfirre-Firre_down	CTAAAAGGATTAGGGTCTCTTGG
5	gRNA_Dxz4_up	CATGCTGCTTTTATGTGCTTCGG
6	gRNA_Dxz4_down	TACTGAAGGAATCGTATGACCGG
7	Primer_fwd_Crossfirre_Crossfirre-Firre	AGAACAGCCCTGGAGGAAAT
8	Primer_rev_Crossfirre	GTAGGCAAGCCTGAGGAAAA
9	Primer_rev_Crossfirre-Firre	CCTGGGTCCTCTATAAAAGCAACAG
10	Primer_fwd_Dxz4	ACAGTGCATCAAAAGCACACG
11	Primer_rev_Dxz4	CCTGGTGGCACAGAACTCTA
12	Primer_rev_Crossfirre_Crossfirre-Firre_WT	TCTCTTGTAAGAGTTCCCATGTGT
13	Primer_fwd_Dxz4_WT	AGTTGGGAGCGAAGCAGAAA

b

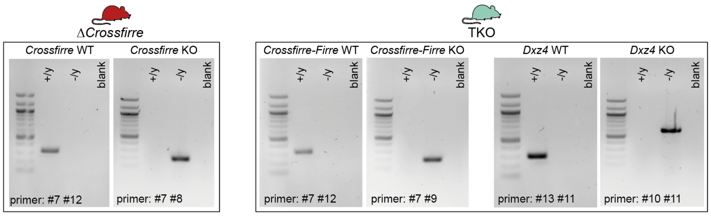

c

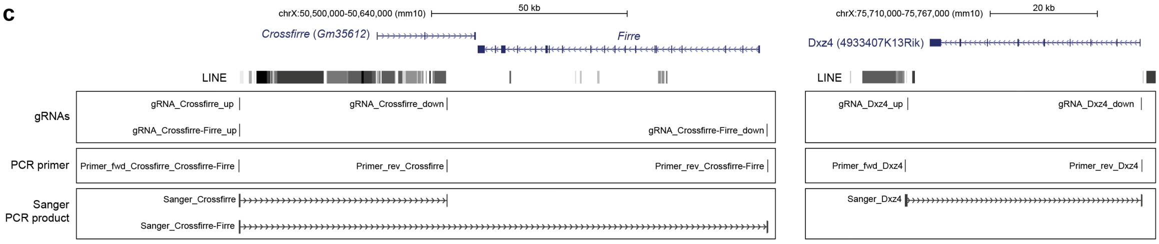

Supplementary Fig. 2: Genotyping and validation of *Crossfirre, Firre,* and *Dxz4* mutants.
a, Required gRNAs for Δ*Crossfirre*, Δ*Crossfirre-Firre*, and Δ*Dxz4* mouse generation and genotyping primers **b**, Genotype strategy for Δ*Crossfirre* and Δ*Crossfirre-Firre-Dxz4* (TKO) to identify the knockout and wildtype allele **c**, UCSC genome browser showing the *Crossfirre-Firre* and *Dxz4* region. The PCR product of the knockout bands was Sanger sequenced, and the resulting sequence was aligned to the UCSC genome browser to confirm the deletion of these loci.

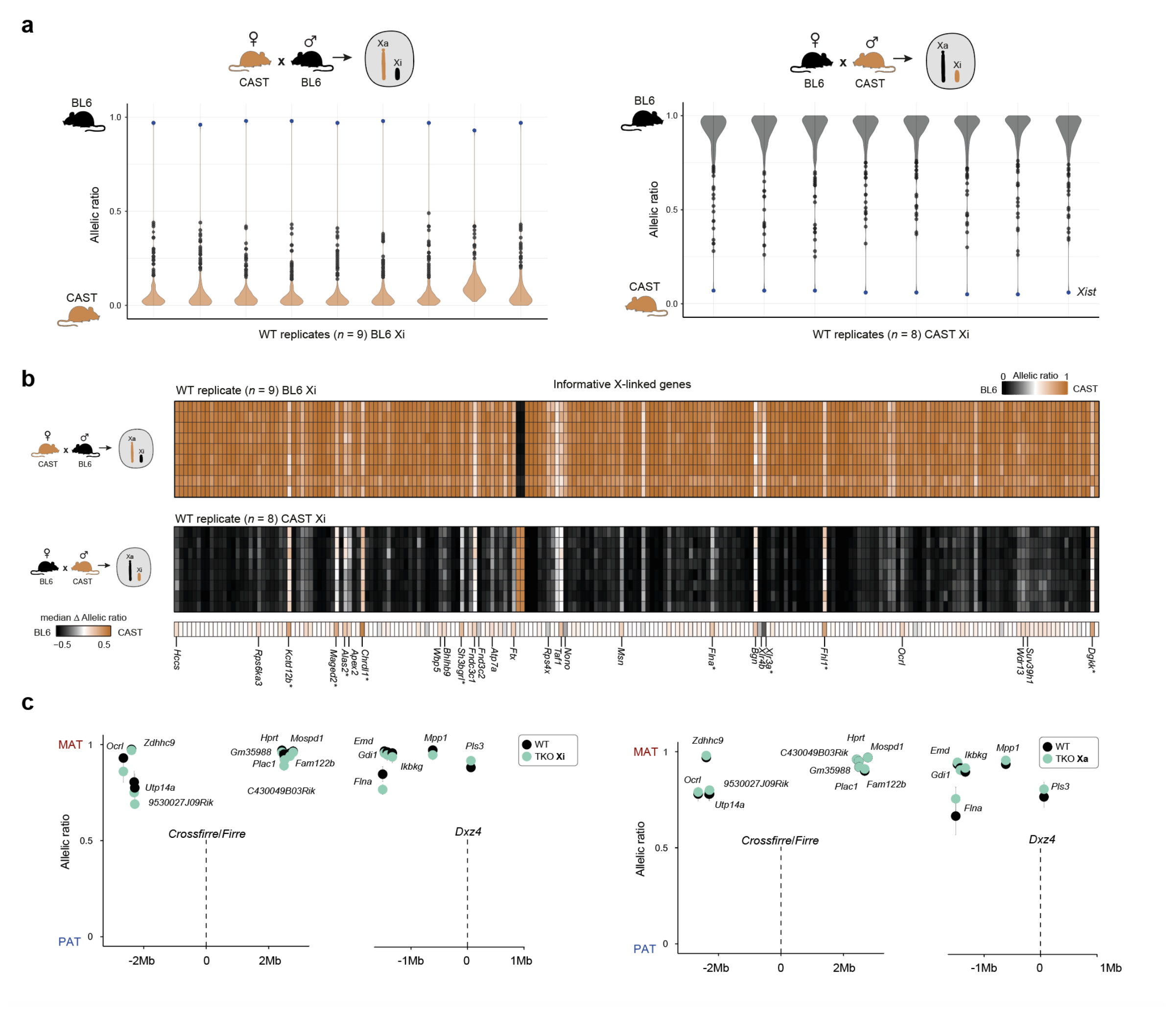

Supplementary Fig. 3: Quality control of allele-specific analysis in placenta and the effect of the TKO on nearby genes.
a, Violin plots of the median allelic ratios for X-linked genes of the wildtype (WT) samples with BL6 Xi (left, *n* = 9) and CAST Xi (right, *n* = 8). Blue dots mark the allelic ratio of *Xist*. **b**, Heatmap showing median allelic ratios for X-linked genes in WT replicates with BL6 Xi (top, *n* = 9) and CAST Xi (bottom, *n* = 8). Genes with median delta allelic ratio ≧ 0.1 between BL6 Xi and CAST Xi samples are highlighted. *Previously reported as strain-specific escapers in [14]. **c**, The median allelic ratios and the standard deviation are shown for genes in the local region of *Crossfirre*/*Firre* (± 2Mb) and *Dxz4* (± 1Mb) for WT (black) and Δ*Crossfirre-Firre-Dxz4* (TKO, turquoise) on Xi (left) or Xa (right, TKO Xi *n* = 3, TKO Xa *n* = 3). Genes with less than 50 SNP-overlapping reads were excluded.

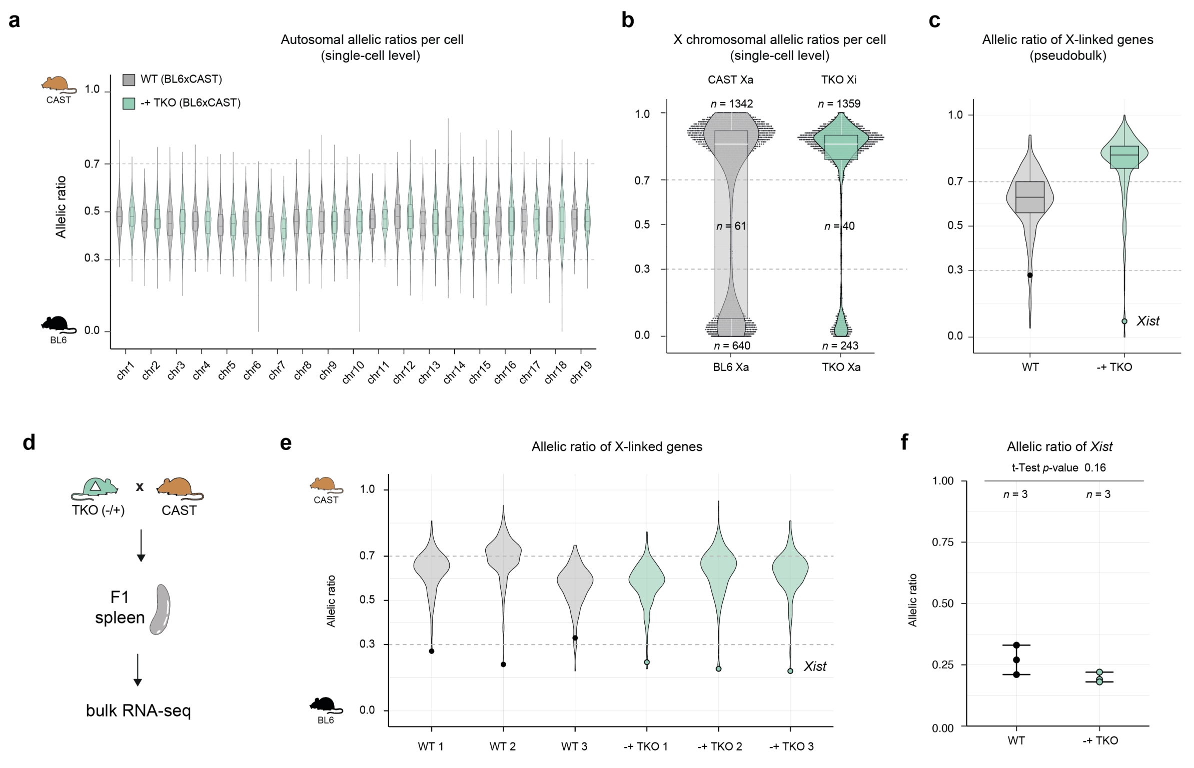

Supplementary Fig. 4: Allele-specific single-cell and bulk RNA-seq analysis of F1 spleens.
a, Violin plots displaying the allelic ratio for each autosome from single-cells of wildtype (WT, *n* = 1, gray) and heterozygous Δ*Crossfirre-Firre-Dxz4* (TKO, *n* = 1, turquoise) spleens in females. **b**, Violin plot of allelic ratios for the X chromosome per cell in WT (gray) and heterozygous TKO (turquoise) spleens. Allelic ratios range from 0 to 1, where 0 corresponds to 100% BL6 Xa and 1 corresponds to 100% CAST Xa. Allelic ratios between 0.3 to 0.7 were classified as biallelic, highlighting cells with both X chromosomes active. **c**, Violin plot of allelic ratios for the X-linked genes. Single-cell reads were combined as pseudobulk for WT (*n* = 1) and heterozygous TKO (*n* = 1) samples. All boxes illustrate the interquartile range around the median, with whiskers extending to 1.5 times the interquartile range. **d**, Schematic workflow showing the experimental setup to further investigate the X chromosome inactivation skewing ratio in WT and heterozygous TKO samples. Heterozygous TKO females (BL6) were mated with WT CAST males to generate F1 hybrids with WT and heterozygous TKO genotypes. Spleens were isolated (*n* = 6) and processed for bulk RNA-seq. **e**, Violin plots showing the allelic ratios of the X-linked genes for each replicate (WT *n* = 3, -/+ TKO *n* = 3). **f**, Allelic ratios plotted for *Xist* per replicate (WT *n* = 3, -/+ TKO *n* = 3). A t-test was used to assess significance between the allelic ratios of WT and heterozygous TKO mutants. The error bars extend from the minimum to the maximum values.

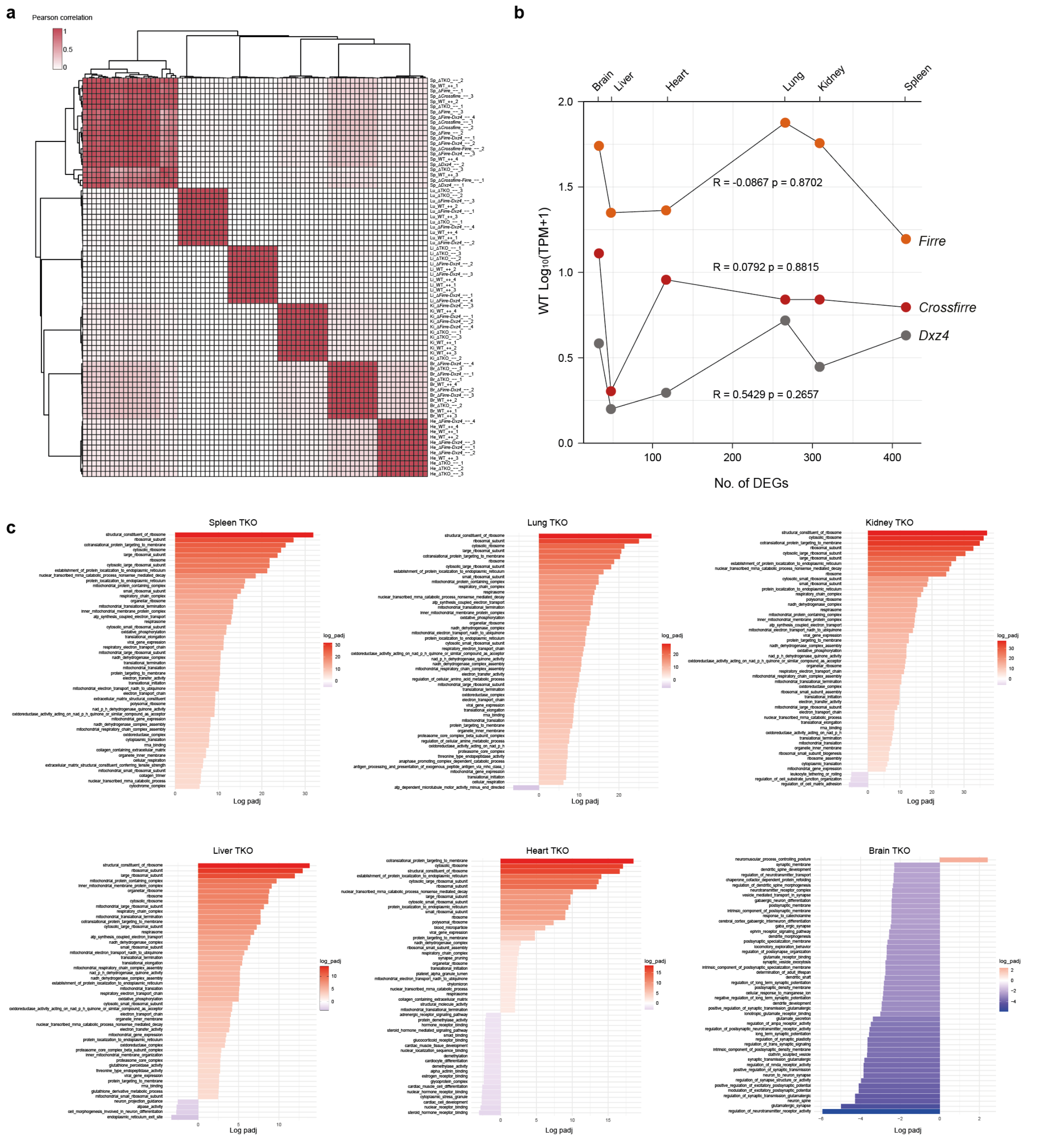

Supplementary Fig. 5: Quality control of the adult transcriptomic bodymap and downstream molecular analysis.
a, Pearson correlation heatmap of the different adult samples included in the bioinformatic analysis (n = 76). The correlation matrix is based on TPM values. **b**, Scatter plot showing the log10-transformed mean TPM+1 correlation between *Crossfirre* (red), *Firre* (orange), and *Dxz4* (gray) and the number of significantly differentially expressed genes in Δ*Crossfirre-Firre-Dxz4* (TKO) samples across the studied tissues (n = 6). Correlations were calculated using Pearson's correlation coefficient. **c**, Dysregulated gene sets of TKO homozygous organs. Top 50 enriched dysregulated gene sets (FDR-adjusted p-value ≤ 0.1) for each bodymap organ of TKO females. The GSEA analysis was performed on DEseq2 test statistics with all gene ontology gene sets (c5.go.v7.4.symbols).

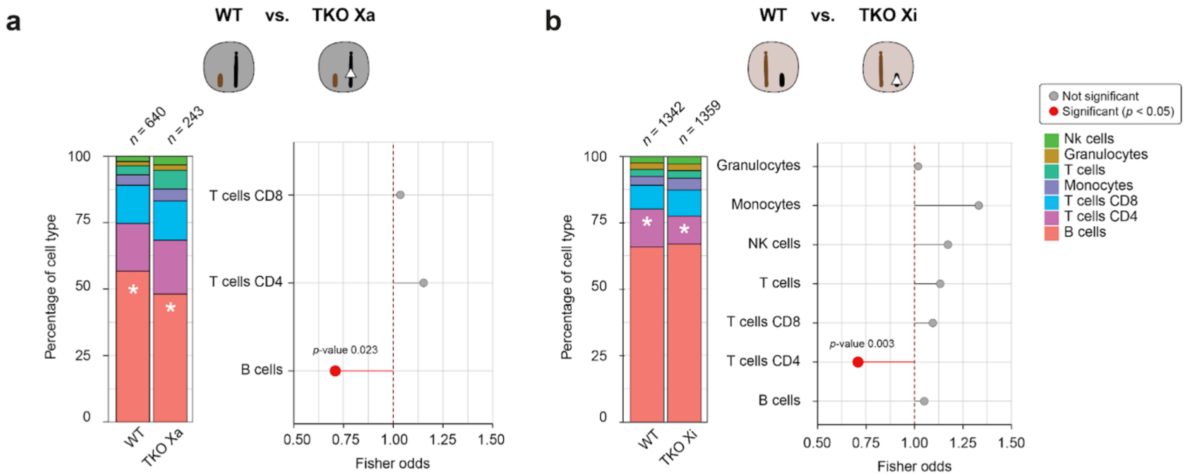

Supplementary Fig. 6: Cell type proportions from scRNA-seq data.
a, Barplot illustrating the distribution of cell types as a percentage derived from scRNA-seq cell counts from wildtype (WT, BL6 Xa) cells and heterozygous ∆*Crossfirre-Firre-Dxz4* (TKO) on Xa. Asterisks indicate statistically significant changes between WT and TKO samples using Fisher's exact test. The right panel shows the odds ratios obtained by Fisher's exact test for cell types containing more than 20 cells, with significant *p*-values highlighted in red. **b**, Same as in **a**, for WT (CAST Xa) cells and heterozygous TKO on Xi.

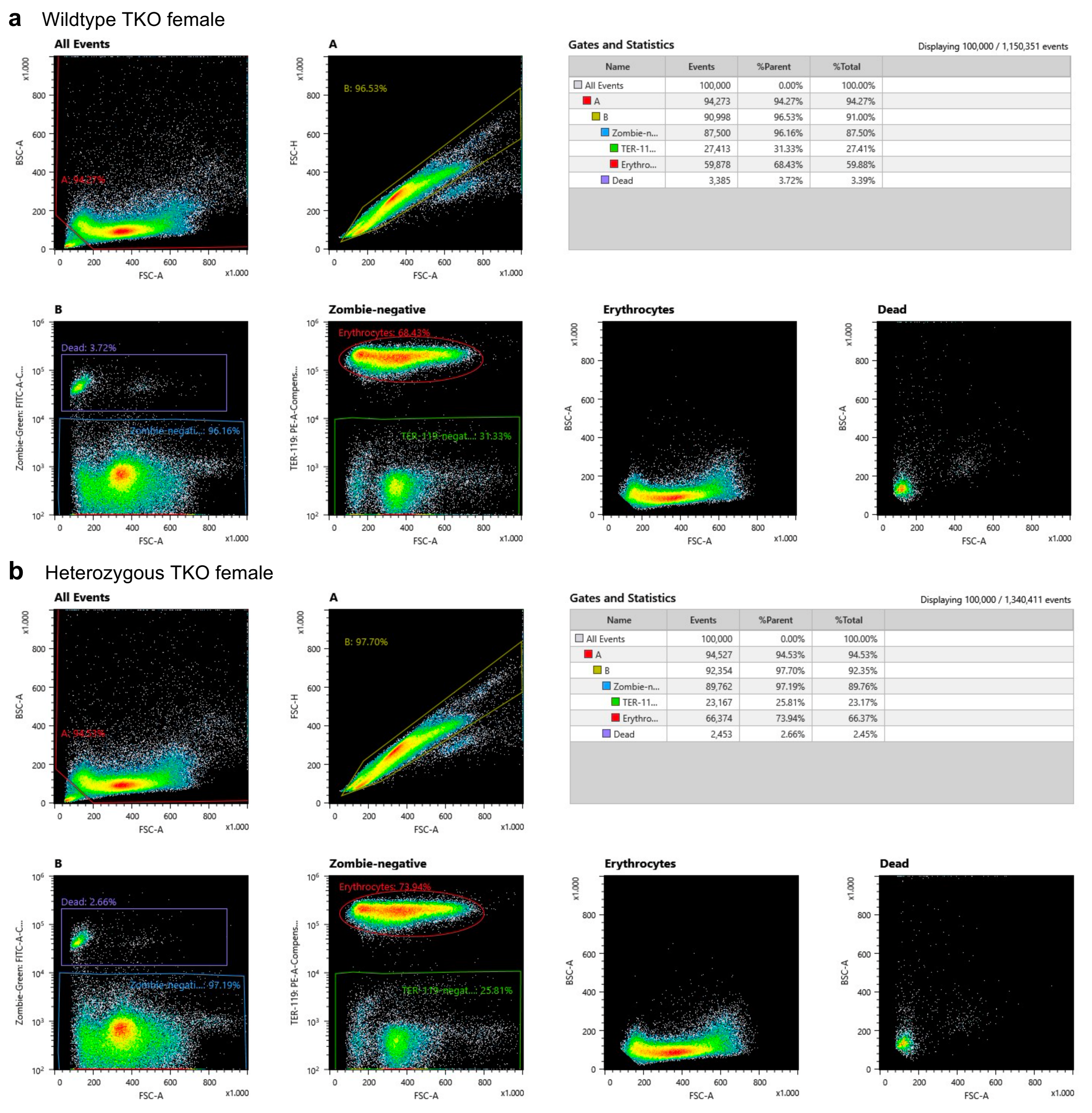

Supplementary Fig. 7: Sorting strategy for single-cell RNA seq experiment

a-b, After single cells of spleens were isolated and stained with Zombie Green for viability and an antibody against TER-119 for erythrocyte detection (see **methods**), cells were sorted by FACS. The first gate excluded debris by considering cells of reasonable size on the FSC-A:BSC-A plot. Subsequently, a gate on the FSC-A:FSC-H plot was employed to exclude doublets. The FSC-A:Zombie-Green:FITC-A plot then gated our sample into dead cells and living cells. The Zombie-Green-negative gate, representing living cells, was visualized on an FSC-A:TER-119:PE-A plot to distinguish between erythrocytes and non-erythrocytes. Non-erythrocytes were sorted for further downstream experiments. Upper panel (**a**) represents the wildtype female, whil lower panel (**b**) represents the heterozygous TKO female.
