# Supplementary material for: X-linked deletion of Crossfirre, Firre, and Dxz4 in vivo uncovers diverse phenotypes and combinatorial effects on autosomes
Source: Nat Commun. 2024 Dec 5;15:10631. doi: 10.1038/s41467-024-54673-5 (PMC11621363; doi:10.1038/s41467-024-54673-5)
Supplement: Supplementary file 3 — Description of Additional Supplementary Files [file 41467_2024_54673_MOESM3_ESM.pdf]

Supplementary Data 1 Multi-omic and phenotypic data from X-linked mutants
a) Sample information from E12.5 F1 embryos
b) Sample information from adult (6 weeks) body map
c) ATAC-seq analysis of male and female organs
d) Oligo information and Sanger sequencing for genotype verification of KO strains
e) Differential gene expression analysis in placenta
f) Allele-specific expression analysis in the placenta
g) Allele-specific single-cell expression analysis in the adult spleen
h) Differential gene expression analysis for the TKO bodymap
i) Significant differentially expressed genes in the TKO and Firre-Dxz4 bodymap
j) Differentially expressed genes in the TKO bodymap
k) Top 100 gene sets from gene set enrichment analysis of the TKO bodymap
l) Differentially expressed genes in the spleen of mutant strains
m) Shared differentially expressed genes in the spleen
n) Gene set enrichment analysis results in the spleen
o-q) German Mouse Clinic TKO phenotype results
r-s) Expression tables (TPM)
