# Supplementary material for: X-linked deletion of Crossfirre, Firre, and Dxz4 in vivo uncovers diverse phenotypes and combinatorial effects on autosomes
Source: Nat Commun. 2024 Dec 5;15:10631. doi: 10.1038/s41467-024-54673-5 (PMC11621363; doi:10.1038/s41467-024-54673-5)
Supplement: Supplementary file 5 — Reporting Summary [file 41467_2024_54673_MOESM5_ESM.pdf]

nature portfolio

Corresponding author(s): Daniel Andergassen

Last updated by author(s): Oct 29, 2024

Reporting Summary

Nature Portfolio wishes to improve the reproducibility of the work that we publish. This form provides structure for consistency and transparency in reporting. For further information on Nature Portfolio policies, see our Editorial Policies and the Editorial Policy Checklist.

Statistics

For all statistical analyses, confirm that the following items are present in the figure legend, table legend, main text, or Methods section.

n/a	Confirmed	
☐	☒	The exact sample size (*n*) for each experimental group/condition, given as a discrete number and unit of measurement
☐	☒	A statement on whether measurements were taken from distinct samples or whether the same sample was measured repeatedly
☐	☒	The statistical test(s) used AND whether they are one- or two-sided<br>*Only common tests should be described solely by name; describe more complex techniques in the Methods section.*
☒	☐	A description of all covariates tested
☐	☒	A description of any assumptions or corrections, such as tests of normality and adjustment for multiple comparisons
☐	☒	A full description of the statistical parameters including central tendency (e.g. means) or other basic estimates (e.g. regression coefficient) AND variation (e.g. standard deviation) or associated estimates of uncertainty (e.g. confidence intervals)
☐	☒	For null hypothesis testing, the test statistic (e.g. *F*, *t*, *r*) with confidence intervals, effect sizes, degrees of freedom and *P* value noted<br>*Give P values as exact values whenever suitable.*
☒	☐	For Bayesian analysis, information on the choice of priors and Markov chain Monte Carlo settings
☒	☐	For hierarchical and complex designs, identification of the appropriate level for tests and full reporting of outcomes
☐	☒	Estimates of effect sizes (e.g. Cohen's *d*, Pearson's *r*), indicating how they were calculated

Our web collection on statistics for biologists contains articles on many of the points above.

Software and code

Policy information about availability of computer code

Data collection	No software was used for data collection.
Data analysis	Samtools (version 1.12)<br>Bowtie (version 2.3.5.1)<br>HTSeq (version 0.11.3)<br>Bedtools (version 2.20.1)<br>Python (version 2.7)<br>R (version ≥ 3.1.0)<br>Perl (version ≥ 5.20.0)<br>GATK (version 3.8)<br>Macs2 (version 2.1.4)<br>Sinto (version 0.8.1)<br>Ucsc-wigtobigwig (version 377)<br>Ucsc-bedtobigbed (version 377)<br>Ucsc-fetchchromsizes (version 377)<br>Deeptools (version 3.3.0)<br>STAR (version 2.6.0c)<br>FastQC (version 0.11.9)<br>Cellranger (version 6.1.2)

SNPsplit (version 0.3.2)
Limma (version 3.58.1)
DESeq2 (version 1.42.0)
Seurat (version 5.0.1)

For manuscripts utilizing custom algorithms or software that are central to the research but not yet described in published literature, software must be made available to editors and reviewers. We strongly encourage code deposition in a community repository (e.g. GitHub). See the Nature Portfolio guidelines for submitting code & software for further information.

Data

Policy information about availability of data

All manuscripts must include a data availability statement. This statement should provide the following information, where applicable:

- Accession codes, unique identifiers, or web links for publicly available datasets
- A description of any restrictions on data availability
- For clinical datasets or third party data, please ensure that the statement adheres to our policy

All generated data have been submitted to the Gene Expression Omnibus (GEO) database under accession code GSE219160.

Research involving human participants, their data, or biological material

Policy information about studies with human participants or human data. See also policy information about sex, gender (identity/presentation), and sexual orientation and race, ethnicity and racism.

Reporting on sex and gender	N/A
Reporting on race, ethnicity, or other socially relevant groupings	N/A
Population characteristics	N/A
Recruitment	N/A
Ethics oversight	N/A

Note that full information on the approval of the study protocol must also be provided in the manuscript.

Field-specific reporting

Please select the one below that is the best fit for your research. If you are not sure, read the appropriate sections before making your selection.

[x] Life sciences   [ ] Behavioural & social sciences   [ ] Ecological, evolutionary & environmental sciences

For a reference copy of the document with all sections, see nature.com/documents/nr-reporting-summary-flat.pdf

Life sciences study design

All studies must disclose on these points even when the disclosure is negative.

Sample size	No sample size calculations were performed. Sample sizes were determined based on previous experiments.
Data exclusions	No data were excluded.
Replication	For the comprehensive bulk RNA sequencing study including the placental samples (n = 53), the TKO female bodymap organs (n = 42) and the Crossfirre SKO spleens, we used at least three biological replicates for each group (3WT vs. 3KO). This number of replicates is generally used for RNA-seq in tissues from inbred mice, as low biological and technical variability between samples is expected. To validate the dysregulated genes from the TKO, we analyzed two replicates from an independently generated female DKO strain. To test the variability in our samples, we performed unsupervised clustering (Pearson correlation) and were able to confirm the identity of the tissues by showing that replicates of the same tissue clustered together. This result implies a low biological and technical variability among the samples, which supports our choice of at least 3 biological replicates.<br>For the single-cell spleen analysis, which we used to investigate the effect of the TKO deletion in a system with random X-chromosome inactivation, we performed an allele-specific analysis using the Allelome.PRO approach, which has the advantage of a higher statistical power because the expression signals of the two alleles serve as internal controls for each other, allowing robust profiling of escapees from a single WT and heterozygous replicate.
Randomization	No randomization was performed.
Blinding	For the in vivo phenotyping of the TKO, blinding was not necessary for most tests, as the experimenter has no influence on the results of these tests, as the results are recorded directly by the machine. For those tests where the experimenter could influence the measurements, the experimenter was blinded.

Reporting for specific materials, systems and methods

We require information from authors about some types of materials, experimental systems and methods used in many studies. Here, indicate whether each material, system or method listed is relevant to your study. If you are not sure if a list item applies to your research, read the appropriate section before selecting a response.

Materials & experimental systems

n/a	Involved in the study
☐	☒ Antibodies
☒	☐ Eukaryotic cell lines
☒	☐ Palaeontology and archaeology
☐	☒ Animals and other organisms
☒	☐ Clinical data
☒	☐ Dual use research of concern
☒	☐ Plants

Methods

n/a	Involved in the study
☒	☐ ChIP-seq
☐	☒ Flow cytometry
☒	☐ MRI-based neuroimaging

Antibodies

Antibodies used	TER-119 Monoclonal Antibody (TER-119), PE, eBioscience™ (Supplier: ThermoFisher, Catalog number:# 12-5921-83, Clone name: TER-119, Lot number: 2252667)
Validation	TER-119 antibody was validated in C57BL/6 mouse bone marrow cells by the supplier. The manufacturer's website states 72 citations for flow cytometry use. (https:/ /www.thermofisher.com/antibody/product/TER-119-Antibody-clone-TER-119-Monoclonal/12-5921-83)

Animals and other research organisms

Policy information about studies involving animals; ARRIVE guidelines recommended for reporting animal research, and Sex and Gender in Research

Laboratory animals	C57BL/6J (BL6), B6D2Fl/J (Fl BL6 and DBA), and CAST/Ei (CAST) mice were purchased from the Jackson Laboratory, and CD-1 females from Charles River. For the adult bodymap, 6-week-old mice were used throughout the entire study. For the imprinted X-inactivation analysis, we collected E12.5 placentas.
Wild animals	This study did not involve wild animals.
Reporting on sex	Since the investigation of sex-specific loci was the major aim of this study, we performed sex-based analyses.
Field-collected samples	This study did not involve samples collected from the field.
Ethics oversight	All procedures have been performed in our specialized facility, followed all relevant animal welfare guidelines and regulations, and were approved by Harvard University IACUC protocol (28-21). Mice were sustained under controlled pathogen-free conditions (Harvard University's Biological Research Infrastructure and Technical University Munich Institute of Pharmacology and Toxicology).

Note that full information on the approval of the study protocol must also be provided in the manuscript.

Plants

Seed stocks	N/A
Novel plant genotypes	N/A
Authentication	N/A

Flow Cytometry

Plots

Confirm that:

- [x] The axis labels state the marker and fluorochrome used (e.g. CD4-FITC).
- [x] The axis scales are clearly visible. Include numbers along axes only for bottom left plot of group (a 'group' is an analysis of identical markers).
- [x] All plots are contour plots with outliers or pseudocolor plots.
- [x] A numerical value for number of cells or percentage (with statistics) is provided.

Methodology

Sample preparation	Spleens from TKO heterozygous females and wildtype littermates were isolated on ice and homogenized between two glass slides. The single-cell suspension was filtered through 70 µm and 30 µm strainers and incubated with Fc-block for 15 min. Then cells were stained with Zombie Green (Viability, BioLegend) and a TER-119-PE antibody (Erythrocytes, ThermoFisher). After staining, cells were incubated with Cell Multiplexing Oligos (CMO, 10X) to add a specific barcode to each sample, allowing the pooling of all four samples in one 10X reaction. The Zombie Green-negative and TER-119-negative population were FACS-sorted and counted for subsequent single-cell library generation.
Instrument	Sony Cell Sorter (make: SONY, Model number: LE-SH800SZGCPL)
Software	SH800 Software
Cell population abundance	Sorted single-cell solution was observed under the microscope and counted by the Countess 3 Automated Cell Counter (Invitrogen) as well as manually. All cells were singlets, no dead cells, no debris was observed
Gating strategy	The first gate excluded debris by considering cells of reasonable size on the FSC-A:BSC-A plot. Subsequently, a gate on the FSC-A:FSC-H plot was employed to exclude doublets. The FSC-A:Zombie-Green:FITC-A plot then gated our sample into dead cells and living cells. The Zombie-Green-negative gate, representing living cells, was visualized on an FSC-A:TER-119:PE-plot to distinguish between Erythrocytes and non-erythrocytes. Non-erythrocytes were sorted for further downstream experiments (10X scRNA-seq). More details are provided in a separate file (Reporting Summary_FACS_info.pdf).

- [x] Tick this box to confirm that a figure exemplifying the gating strategy is provided in the Supplementary Information.
